# Supplementary material for: Incidence and Survival Trends of Pancreatic Cancer in Girona: Impact of the Change in Patient Care in the Last 25 Years
Source: Int J Environ Res Public Health. 2020 Dec 19;17(24):9538. doi: 10.3390/ijerph17249538 (PMC7766657; doi:10.3390/ijerph17249538)
Supplement: Supplementary file 1 [file ijerph-17-09538-s001.pdf]

a) Both sexes

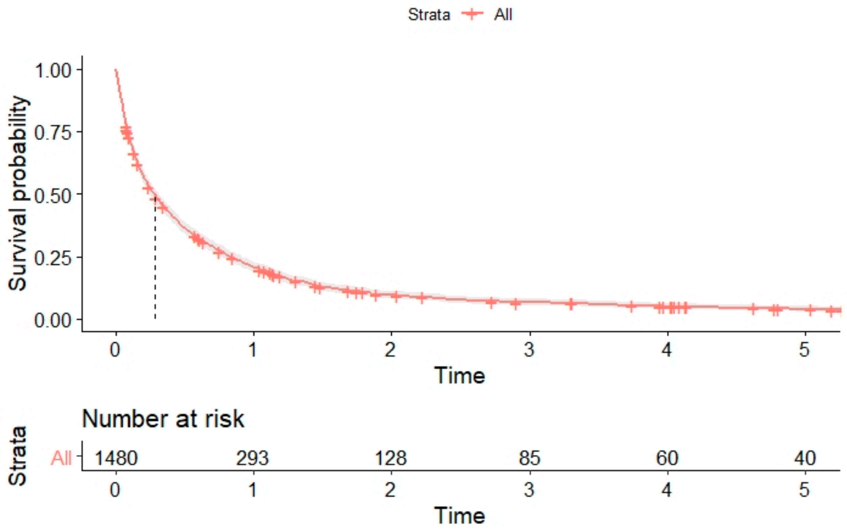

b) Male (blue line) Females (red line)

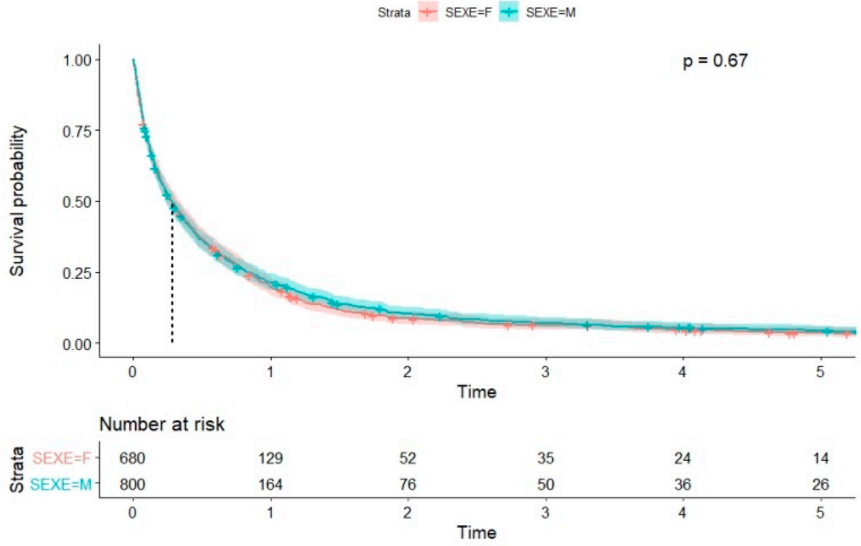

c) By histology: Non PNETs and non-histologically confirmed PC (blue lines, NonNed), and PNETs (red lines, NEd).

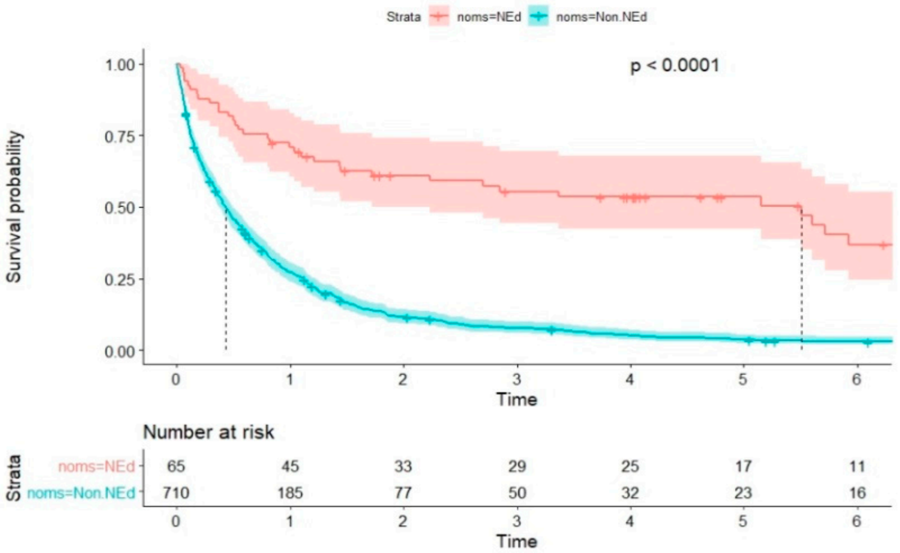

Figure S1. Observed survival curves (%) up to 5 years after diagnosis. a) both sexes b) men and women c) histology groups.

Table S1. Frequency of the histological groupings based on the classification of pancreatic tumours of the 4th edition of *WHO classification of tumours of the digestive system*. Histologies present in our study that are not found on the classification are included in the table.

CCode	HISTOLOGY	Cases during 1994-2015 n (%)
	**Pancreatic carcinomas non-neuroendocrine**	**711 (44.38)**
8010/3	Carcinoma, NOS	39 (2.4)
8020/3	Carcinoma, undifferentiated, NOS	4 (0.2)
8035/3	Carcinoma with osteoclast-like giant cells	1 (0.1)
8070/3	Squamous cell carcinoma, NOS	1 (0.1)
8140/3	Adenocarcinoma, NOS	471 (29.4)
8260/3	Papillary adenocarcinoma, NOS	2 (0.1)
8452/3	Solid pseudopapillary carcinoma	1 (0.1)
8453/3	Intraductal papillary-mucinous carcinoma, invasive	3 (0.2)
8470/3	Mucinous cystadenocarcinoma, NOS	2 (0.1)
8480/3	Mucinous adenocarcinoma	5 (0.3)
8481/3	Mucin-producing adenocarcinoma	13 (0.8)
8490/3	Signet ring cell carcinoma	3 (0.2)
8500/3	Infiltrating duct carcinoma, NOS	156 (9.7)
8510/3	Medullary carcinoma, NOS	1 (0.1)
8550/3	Acinar cell carcinoma	7 (0.4)
8560/3	Adenosquamous carcinoma	2 (0.1)
8971/3	Pancreatoblastoma	1 (0.1)
	**Pancreatic neuroendocrine cancers**	**65 (4.05)**
8041/3	Small cell carcinoma, NOS	5 (0.3)
8150/3	Pancreatic endocrine tumour, malignant	23 (1.4)
8151/3	Insulinoma, malignant	8 (0.5)
8153/3	Gastrinoma, malignant	3 (0.2)
8240/3	Carcinoid tumour, NOS	7 (0.4)
8246/3	Neuroendocrine carcinoma, NOS	18 (1.1)
8249/3	Atypical carcinoid tumour	1 (0.1)
	**Pancreatic tumors non-histologically confirmed**	**825 (51.5)**
8000/3	Malignant neoplasm	825 (51.5)
	**Total**	**1602 (100.0)**

Table S2. Pancreatic cancer rates for each individual year (1994-2015). Total incidence of pancreatic cancer in the province of Girona during the period 1994–2015. The table shows the incidence for each year of the time period studied and the total. Incidence is shown as crude rate (CR) and adjusted by Europe age rates 1976 (ASR_{E76}), Europe 2013 (ASR_{E13}), United States (ASR_{USA}) and World rates (ASR_{W}). The total cases per year and population are also displayed. ASR—Age-standardized rate. CR—Crude rate.

Year	N (number of cases)	Population	CR	ASR_{E76}	ASR_{E13}	ASR_{USA}	ASR_{W}
1994	45	521304	8.63 (6.11–11.15)	6.55 (4.68–9.12)	10.68 (7.76–14.45)	7.59 (5.5–10.35)	4.21 (2.94–6.45)
1995	50	524871	9.53 (6.89–12.17)	7.37 (5.4–10.05)	11.62 (8.59–15.49)	8.29 (6.11–11.13)	5 (3.58–7.37)
1996	39	528716	7.38 (5.06–9.69)	5.77 (4.06–8.19)	8.79 (6.22–12.17)	5.92 (4.18–8.31)	3.96 (2.75–6.17)
1997	52	530505	9.8 (7.14–12.47)	7.66 (5.63–10.39)	11.7 (8.7–15.47)	8.18 (6.07–10.95)	5.18 (3.76–7.57)
1998	53	535077	9.91 (7.24–12.57)	7.41 (5.47–10.04)	11.49 (8.58–15.16)	7.93 (5.91–10.59)	4.97 (3.62–7.29)
1999	50	540468	9.25 (6.69–11.82)	6.72 (4.88–9.24)	11.11 (8.22–14.73)	7.74 (5.72–10.4)	4.29 (3.05–6.52)
2000	71	546755	12.99 (9.97–16.01)	9.57 (7.39–12.42)	14.66 (11.43–18.57)	10.11 (7.86–12.96)	6.51 (4.96–8.97)
2001	71	556891	12.75 (9.78–15.71)	9.24 (7.1–12.02)	14.37 (11.2–18.19)	10.22 (7.96–13.1)	6.2 (4.68–8.61)
2002	66	574342	11.49 (8.72–14.26)	8.49 (6.44–11.16)	13.11 (10.12–16.76)	9.13 (7.04–11.81)	5.79 (4.31–8.11)
2003	69	594461	11.61 (8.87–14.35)	8.21 (6.27–10.73)	13.05 (10.14–16.61)	9.16 (7.11–11.78)	5.34 (4.01–7.47)
2004	85	616994	13.78 (10.85–16.71)	9.79 (7.72–12.41)	16.02 (12.78–19.88)	10.86 (8.66–13.61)	6.48 (5.02–8.65)
2005	64	643599	9.94 (7.51–12.38)	7.53 (5.7–9.88)	11.37 (8.74–14.59)	7.91 (6.08–10.27)	5.13 (3.82–7.13)
2006	68	667724	10.18 (7.76–12.6)	7.81 (5.96–10.18)	11.54 (8.95–14.71)	8.35 (6.47–10.75)	5.31 (3.99–7.29)
2007	83	695361	11.94 (9.37–14.5)	8.93 (7–11.35)	14.13 (11.23–17.61)	9.72 (7.73–12.21)	6.09 (4.7–8.08)
2008	75	720204	10.41 (8.06–12.77)	8.12 (6.28–10.45)	12.36 (9.7–15.57)	8.69 (6.82–11.04)	5.35 (4.08–7.2)
2009	78	737621	10.57 (8.23–12.92)	8.13 (6.35–10.38)	12.9 (10.17–16.17)	8.76 (6.91–11.07)	5.49 (4.22–7.32)
2010	89	744485	11.95 (9.47–14.44)	9.21 (7.3–11.57)	14.13 (11.32–17.48)	9.77 (7.83–12.17)	6.17 (4.83–8.04)
2011	96	749509	12.81 (10.25–15.37)	9.42 (7.53–11.75)	14.82 (11.97–18.2)	10.3 (8.33–12.72)	6.34 (5.01–8.2)
2012	103	751279	13.71 (11.06–16.36)	10.16 (8.19–12.57)	15.72 (12.79–19.17)	10.82 (8.81–13.27)	6.95 (5.54–8.89)
2013	109	748341	14.57 (11.83–17.3)	10.38 (8.41–12.77)	16.09 (13.17–19.53)	11.36 (9.3–13.85)	6.86 (5.49–8.75)
2014	89	743352	11.97 (9.49–14.46)	8.53 (6.77–10.73)	13.31 (10.64–16.48)	9.38 (7.51–11.69)	5.81 (4.56–7.63)
2015	97	738976	13.13 (10.51–15.74)	9.28 (7.44–11.57)	14.21 (11.49–17.43)	9.91 (8.01–12.25)	6.15 (4.87–8)
Total	1602	14010835	11.43 (10.87–11.99)	8.47 (8.04–8.92)	13.19 (12.55–13.85)	9.19 (8.75–9.66)	5.68 (5.38–6.01)

Table S3. Pancreatic cancer rates for each individual year (1994-2015). Incidence of pancreatic cancer in men in the province of Girona during the period 1994–2015. The table shows the incidence for each year and the total. Incidence is shown as crude rate (CR) and adjusted by Europe age rates 1976 (ASR_{E76}), Europe 2013 (ASR_{E13}), United States (ASR_{USA}) and World rates (ASR_{W}). The total cases per year and population are also displayed. ASR—Age-standardized rate. CR—Crude rate.

Year	N (number of cases)	Population	CR	ASR_{E76}	ASR_{E13}	ASR_{USA}	ASR_{W}
1994	20	258490	7.74 (4.35–11.13)	7.25 (4.35–11.68)	10.44 (6.27–16.99)	7.6 (4.57–12.19)	4.91 (2.9–9.01)
1995	27	260105	10.38 (6.46–14.3)	8.84 (5.78–13.35)	14.33 (9.25–21.74)	10.01 (6.49–15.03)	5.93 (3.81–10.07)
1996	24	261866	9.16 (5.5–12.83)	7.76 (4.93–12.07)	11.59 (7.34–18.01)	7.81 (4.93–12.07)	5.33 (3.34–9.39)
1997	31	262541	11.81 (7.65–15.96)	10.05 (6.76–14.79)	15.65 (10.45–22.98)	10.86 (7.27–15.84)	6.78 (4.51–11.07)
1998	26	264805	9.82 (6.04–13.59)	7.75 (5.02–11.92)	12.69 (8.16–19.28)	8.39 (5.4–12.75)	5.16 (3.29–9.11)
1999	25	267535	9.34 (5.68–13.01)	7.93 (5.06–12.26)	13.23 (8.41–20.11)	9.09 (5.82–13.76)	5.03 (3.14–9)
2000	39	270982	14.39 (9.88–18.91)	12.02 (8.48–16.95)	17.49 (12.33–24.48)	12.22 (8.62–17.12)	8.4 (5.86–12.83)
2001	42	276397	15.2 (10.6–19.79)	12.38 (8.82–17.28)	19 (13.6–26.23)	13.79 (9.89–18.99)	8.37 (5.84–12.7)
2002	33	285504	11.56 (7.61–15.5)	9.33 (6.3–13.66)	14.79 (10.14–21.22)	10.35 (7.09–14.86)	6.38 (4.19–10.34)
2003	38	296580	12.81 (8.74–16.89)	10.34 (7.2–14.7)	16.8 (11.8–23.52)	11.66 (8.21–16.32)	6.53 (4.45–10.27)
2004	45	308668	14.58 (10.32–18.84)	12.54 (9.05–17.17)	18.98 (13.75–25.84)	13.06 (9.48–17.79)	8.59 (6.12–12.57)
2005	39	323703	12.05 (8.27–15.83)	10.1 (7.09–14.19)	15.16 (10.74–21.15)	10.48 (7.44–14.62)	6.85 (4.73–10.4)
2006	42	337283	12.45 (8.69–16.22)	10.94 (7.81–15.13)	15.57 (11.18–21.46)	11.07 (7.96–15.25)	7.63 (5.38–11.21)
2007	44	351751	12.51 (8.81–16.2)	10.96 (7.88–15.06)	17.14 (12.37–23.37)	11.72 (8.49–16)	7.47 (5.3–10.9)
2008	39	365578	10.67 (7.32–14.02)	9.91 (6.99–13.83)	14.22 (10.04–19.78)	10.09 (7.16–14.05)	6.75 (4.71–9.99)
2009	37	374797	9.87 (6.69–13.05)	8.65 (6.04–12.22)	13.57 (9.5–18.97)	9.15 (6.43–12.86)	5.96 (4.1–9)
2010	56	377090	14.85 (10.96–18.74)	12.76 (9.56–16.88)	19.55 (14.68–25.64)	13.66 (10.3–17.97)	8.52 (6.31–11.81)
2011	52	378758	13.73 (10–17.46)	11.41 (8.44–15.29)	17.65 (13.11–23.37)	12.33 (9.2–16.39)	7.69 (5.62–10.84)
2012	59	378628	15.58 (11.61–19.56)	12.98 (9.81–17.05)	19.49 (14.76–25.36)	13.58 (10.32–17.74)	9.18 (6.87–12.57)
2013	50	375944	13.3 (9.61–16.99)	11.01 (8.12–14.8)	16.45 (12.14–21.92)	11.6 (8.6–15.52)	7.41 (5.4–10.52)
2014	42	372502	11.28 (7.87–14.69)	8.84 (6.32–12.26)	13.95 (10–19.02)	9.55 (6.87–13.15)	5.95 (4.19–8.89)
2015	55	369555	14.88 (10.95–18.82)	11.83 (8.85–15.7)	17.95 (13.47–23.55)	12.41 (9.33–16.41)	7.79 (5.77–10.99)
Total	865	7019062	12.32 (11.5–13.14)	10.34 (9.65–11.08)	15.8 (14.75–16.91)	11 (10.27–11.77)	7 (6.51–7.54)

Table S4. Pancreatic cancer rates for each individual year (1994-2015). Incidence of pancreatic cancer in women in the province of Girona during the period 1994–2015. The table shows the incidences per year and the total. Incidence is shown as crude rate (CR) and adjusted by Europe age rates 1976 (ASR_{E76}), Europe 2013 (ASR_{E13}), United States (ASR_{USA}) and World rates(ASR_{W}).. The total cases per year and population are also displayed. ASR—Age-standardized rate. CR—Crude rate.

Year	N (number of cases)	Population	CR	ASR_{E76}	ASR_{E13}	ASR_{USA}	ASR_{W}
1994	25	262814	9.51 (5.78–13.24)	5.57 (3.48–9.09)	10.26 (6.62–15.42)	7.11 (4.57–10.97)	3.37 (2.01–7.01)
1995	23	264766	8.69 (5.14–12.24)	6.25 (3.79–10.22)	9.67 (6.1–14.72)	7.03 (4.4–11.03)	4.25 (2.46–8.29)
1996	15	266850	5.62 (2.78–8.47)	3.96 (2.15–7.34)	6.24 (3.48–10.49)	4.2 (2.33–7.47)	2.7 (1.42–6.4)
1997	21	267964	7.84 (4.48–11.19)	5.67 (3.35–9.54)	8.65 (5.33–13.48)	6.07 (3.7–9.81)	3.8 (2.17–7.71)
1998	27	270272	9.99 (6.22–13.76)	7.32 (4.64–11.51)	10.78 (7.08–15.96)	7.8 (5.07–11.86)	4.94 (3.06–9.04)
1999	25	272933	9.16 (5.57–12.75)	5.79 (3.55–9.52)	9.65 (6.22–14.48)	6.78 (4.34–10.52)	3.7 (2.18–7.51)
2000	32	275773	11.6 (7.58–15.62)	7.18 (4.76–11.01)	11.93 (8.14–17.02)	8.05 (5.46–11.89)	4.66 (2.99–8.5)
2001	29	280494	10.34 (6.58–14.1)	6.41 (4.13–10.06)	10.51 (7.02–15.29)	7.15 (4.75–10.82)	4.18 (2.6–7.88)
2002	33	288838	11.43 (7.53–15.32)	7.82 (5.18–11.77)	11.87 (8.13–16.9)	8.27 (5.64–12.13)	5.29 (3.41–9.09)
2003	31	297881	10.41 (6.74–14.07)	6.51 (4.26–10)	10.35 (7.01–14.95)	7.35 (4.95–10.97)	4.37 (2.77–7.83)
2004	40	308326	12.97 (8.95–16.99)	6.96 (4.84–10.18)	12.84 (9.15–17.69)	8.5 (6.05–12.05)	4.31 (2.91–7.41)
2005	25	319896	7.82 (4.75–10.88)	5.17 (3.19–8.29)	8.01 (5.15–12.02)	5.62 (3.6–8.77)	3.52 (2.09–6.6)
2006	26	330441	7.87 (4.84–10.89)	4.76 (2.91–7.71)	7.58 (4.92–11.32)	5.65 (3.64–8.78)	3.05 (1.78–5.92)
2007	39	343610	11.35 (7.79–14.91)	7.08 (4.83–10.37)	11.53 (8.14–16)	7.97 (5.63–11.36)	4.82 (3.17–7.84)
2008	36	354626	10.15 (6.84–13.47)	6.22 (4.12–9.34)	10.17 (7.08–14.33)	7.04 (4.88–10.22)	3.89 (2.48–6.64)
2009	41	362824	11.3 (7.84–14.76)	7.59 (5.27–10.9)	12.12 (8.64–16.65)	8.3 (5.91–11.68)	5.02 (3.39–7.89)
2010	33	367395	8.98 (5.92–12.05)	6 (3.98–8.99)	9.64 (6.59–13.74)	6.46 (4.41–9.49)	3.97 (2.56–6.64)
2011	44	370751	11.87 (8.36–15.37)	7.53 (5.29–10.71)	12.28 (8.84–16.72)	8.47 (6.09–11.78)	5.05 (3.45–7.84)
2012	44	372651	11.81 (8.32–15.3)	7.38 (5.16–10.52)	12.08 (8.7–16.47)	8.12 (5.85–11.32)	4.73 (3.23–7.44)
2013	59	372397	15.84 (11.8–19.89)	9.56 (7.03–13.01)	15.24 (11.51–19.95)	10.77 (8.12–14.34)	6.22 (4.47–9.15)
2014	47	370850	12.67 (9.05–16.3)	8.32 (5.95–11.61)	12.83 (9.35–17.3)	9.32 (6.79–12.81)	5.74 (4.01–8.74)
2015	42	369421	11.37 (7.93–14.81)	6.81 (4.71–9.85)	10.54 (7.53–14.47)	7.47 (5.31–10.58)	4.56 (3.06–7.4)
Total	737	6991773	10.54 (9.78–11.3)	6.73 (6.21–7.29)	10.82 (10.05–11.65)	7.54 (7–8.13)	4.44 (4.07–4.86)
